# Metastasis of lung carcinoma in a pheochromocytoma

**DOI:** 10.1210/jcemcr/luag283

**Published:** 2026-09-25

**Authors:** Antonio Gigante, Erika Messina, Federica Massa, Marco Volante, Anna Pia, Massimo Terzolo

**Affiliations:** Internal Medicine, Department of Clinical and Biological Sciences, San Luigi Gonzaga Hospital, University of Turin, Orbassano 10043, Italy; Internal Medicine, Department of Clinical and Biological Sciences, San Luigi Gonzaga Hospital, University of Turin, Orbassano 10043, Italy; Pathology Unit and Department of Oncology, San Luigi Gonzaga Hospital, University of Turin, Orbassano 10043, Italy; Pathology Unit and Department of Oncology, San Luigi Gonzaga Hospital, University of Turin, Orbassano 10043, Italy; Department of Endocrinology, Diabetology and Metabolism, A.O. Santa Croce e Carle, Cuneo 12100, Italy; Internal Medicine, Department of Clinical and Biological Sciences, San Luigi Gonzaga Hospital, University of Turin, Orbassano 10043, Italy

**Keywords:** tumor-to-tumor metastasis, lung carcinoma, pheochromocytoma, PPGL, multiple neoplasms, histopathology, immunohistochemistry

## Abstract

Tumor-to-tumor metastasis (TTM) is an established but rare entity. Lung carcinoma metastasizes commonly to adrenal glands; however, metastases into an already existing adrenal gland tumor are a rare finding. A 78-year-old man was diagnosed with pheochromocytoma, and during the disease staging, lung involvement was revealed. Functional imaging with both ^18^F-fluorodeoxyglucose (^18^F-FDG) and ^68^Ga-DOTA-Tyr3-Octreotate (^68^Ga-DOTA-TATE) positron emission tomography (PET) was performed: ^18^FDG-PET showed mild uptake in both the adrenal and pulmonary lesions, whereas ^68^Ga-DOTA-TATE-PET revealed intense uptake confined to the adrenal mass. This discordant pattern suggested the presence of 2 biologically distinct lesions. The right adrenal gland was surgically removed, and histology and immunohistochemistry confirmed 2 components within the tumor: a pheochromocytoma that included a lung cancer metastasis. Histopathological confirmation of the pulmonary primary lesion was not available. This is the second published case of a TTM of lung carcinoma inside a pheochromocytoma. Tumor-to-tumor metastasis should be considered in pathological diagnosis.

## Introduction

Tumor-to-tumor metastasis (TTM) is a rare event. The tumors that most commonly metastasize in other tumors are breast and lung carcinoma, while the tumors that most frequently host TTM are renal cell carcinoma and meningioma [1-3]. Pheochromocytoma is infrequently involved in TTM [4, 5], while the adrenal glands are the fifth site of metastasis from lung carcinoma [6].

We present a case of TTM caused by a lung cancer metastasis in a pheochromocytoma. To our knowledge, this is the second case after the first report in 2009 [7].

## Case presentation

A 78-year-old man with a 30-year history of hypertension, smoking, type 2 diabetes, and stage IV chronic kidney disease, and a family history notable for both parents having died from malignancies of unspecified origin, underwent a transurethral resection for a noninvasive bladder tumor in 2013. At that time, a computed tomography (CT) scan revealed a nodule in the right adrenal gland. The radiologic report did not give more details. The patient was followed for bladder cancer at another institution, without evidence of clinically significant abnormalities during the available follow-up.

In August 2021, an abdominal ultrasound performed during routine follow-up revealed a 42 × 37 mm mass of uncertain nature near the right renal hilum.

**Figure 1:**
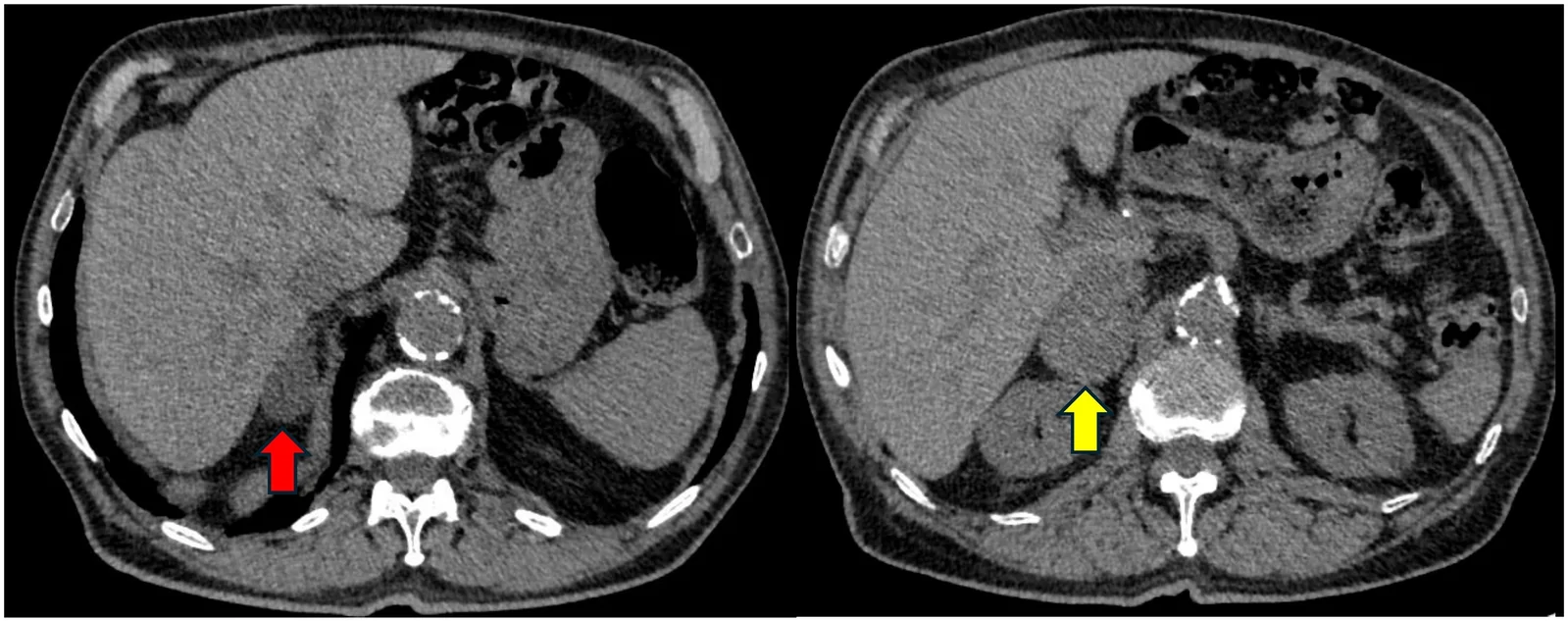
Unenhanced CT of right adrenal gland lesions. Unenhanced abdominal CT scan showing 2 nodular lesions in the right adrenal gland: a 31 × 20 mm low-density lesion (HU close to 0), consistent with an adenoma (left arrow), and a 44 × 38 mm high-density lesion (30-40 HU) located at the inferior pole of the gland (right arrow).

An unenhanced abdomen CT scan showed 2 nodular lesions in the right adrenal gland: a 25 mm low-density lesion [Hounsfield Unit (HU) close to 0], suggestive of adenoma, and a 40 mm high-density lesion (47 HU) located at the lower pole of the gland (Fig. 1). The latter was described in the radiology report as a possible encapsulated hematoma.

## Diagnostic assessment

In October 2021, urinary normetanephrine level was markedly elevated at 3270 μg/24 h (SI: 17 848 nmol/24 h) (reference range 82-424 μg/24 h; [SI: 448-2313 nmol/24 h]), while urinary metanephrine level was modestly elevated at 440 μg/24 h (SI: 2231 nmol/24 h) (reference range 34-240 μg/24 h; [SI: 172-1217 nmol/24 h]). Urinary 3-methoxytyramine (3-MT) was within the normal range at 463 nmol/24 h (reference range 441-394 nmol/24 h). Plasma metanephrine and normetanephrine levels were subsequently found to be markedly elevated at 1311 pmol/L (reference range <300 pmol/L) and 23 426 pmol/L (reference range <600 pmol/L), respectively. Initially, endocrine work-up excluded hypercortisolism, as well as abnormalities in the renin-aldosterone axis and androgen profile.

Biochemical findings were consistent with pheochromocytoma. Therefore, treatment with doxazosine 3 mg/day was started and both a ^18^F-fluorodeoxyglucose (^18^FDG) and a ^68^Ga-DOTA-Tyr3-Octreotate (^68^Ga-DOTA-TATE) positron emission tomography (PET) were scheduled.

In November 2021, ^18^FDG-PET demonstrated increased tracer uptake in the right upper abdomen and chest. Axial images at the level of the right adrenal gland showed a nodular lesion with increased metabolic activity [maximum standardized uptake value (SUVmax) of 4.6]. An additional focus of increased uptake (SUVmax 3.3) was detected in the upper lobe of the right lung, raising suspicion for a neoplastic lesion.

**Figure 2:**
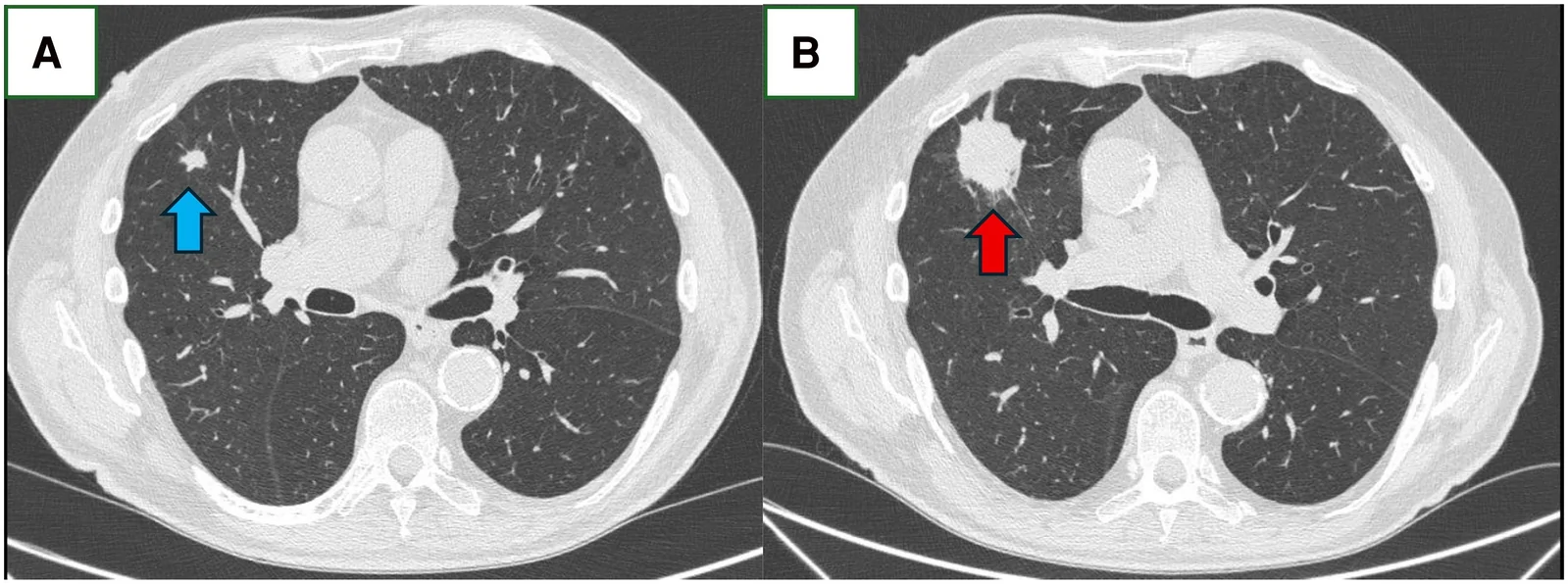
High-resolution CT of right pulmonary nodule. (A) High-resolution chest CT showing an irregular pulmonary nodule measuring 12 × 15 × 17 mm in the right lung (arrow). (B) High-resolution chest CT performed after 3 months showing enlargement of the pulmonary nodule to 44 × 32 × 35 mm (arrow).

To further characterize the area of increased uptake, a high-resolution computed tomography (HRCT) scan of the chest was performed. The scan confirmed the presence of an irregular pulmonary nodule measuring 12 × 15 × 17 mm (Fig. 2A), as well as the 2 previously known nodules in the right adrenal gland, which were slightly increased in size, but with unchanged radiological features.

**Figure 3:**
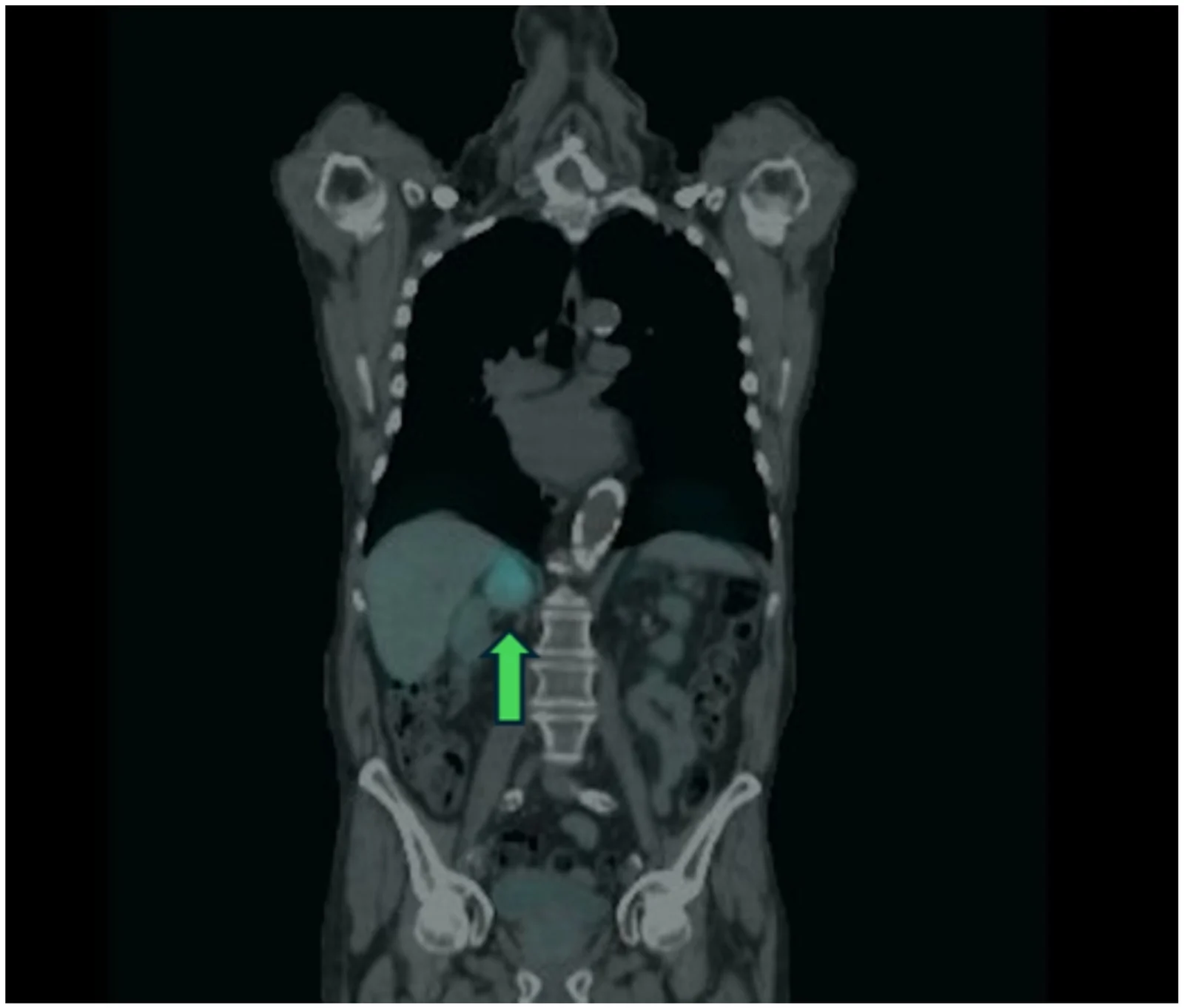
Functional PET/CT evaluation. Coronal ^68^Ga-DOTA-TATE PET image showing increased uptake confined to the right adrenal gland (arrow).

Subsequently, the ^68^Ga-DOTA-TATE-PET was performed in December 2021 and, surprisingly, it showed intense uptake confined to the right adrenal gland (SUVmax 26.94) (Fig. 3).

## Treatment

Following multidisciplinary discussion and considering the persistent risk of catecholamine-related hemodynamic instability despite alpha-blockade, procedural risk, the patient's age, comorbidities, and preference, a wait-and-see approach was adopted. After 3 months the CT scan showed enlargement of the right lung nodule to 44 × 32 × 35 mm (Fig. 2B). Given its radiological features and the history of heavy smoking, the lesion was managed as a primary lung cancer. Considering the anesthetic risks associated with 2 surgical procedures, radiotherapy (RT) was initiated and right adrenalectomy was scheduled after completion of RT.

The patient received 60 Gy in 8 fractions, resulting in a dimensional decrease of the lung nodule. During treatment, blood pressure (BP) remained well controlled, with an average home measurement of 120/75 mmHg. In January 2023, right adrenalectomy was performed after a new abdominal CT scan which demonstrated a slight increase in the size of the hyperdense right adrenal nodule to 55 × 55 × 55 mm. Preoperative preparation included infusion of 750 mL of 0.9% saline solution during the 24 hours before surgery, while doxazosin 3 mg/day, initiated after biochemical diagnosis, was continued until 12 hours before surgery.

## Outcome and follow-up

Throughout the perioperative period, the patient remained hemodynamically stable, and the postoperative course was uneventful. Following surgery, he discontinued doxazosine maintaining a suboptimal BP control (average home measurement of 150/70 mmHg); thus, amlodipine 10 mg/day was added.

**Figure 4:**
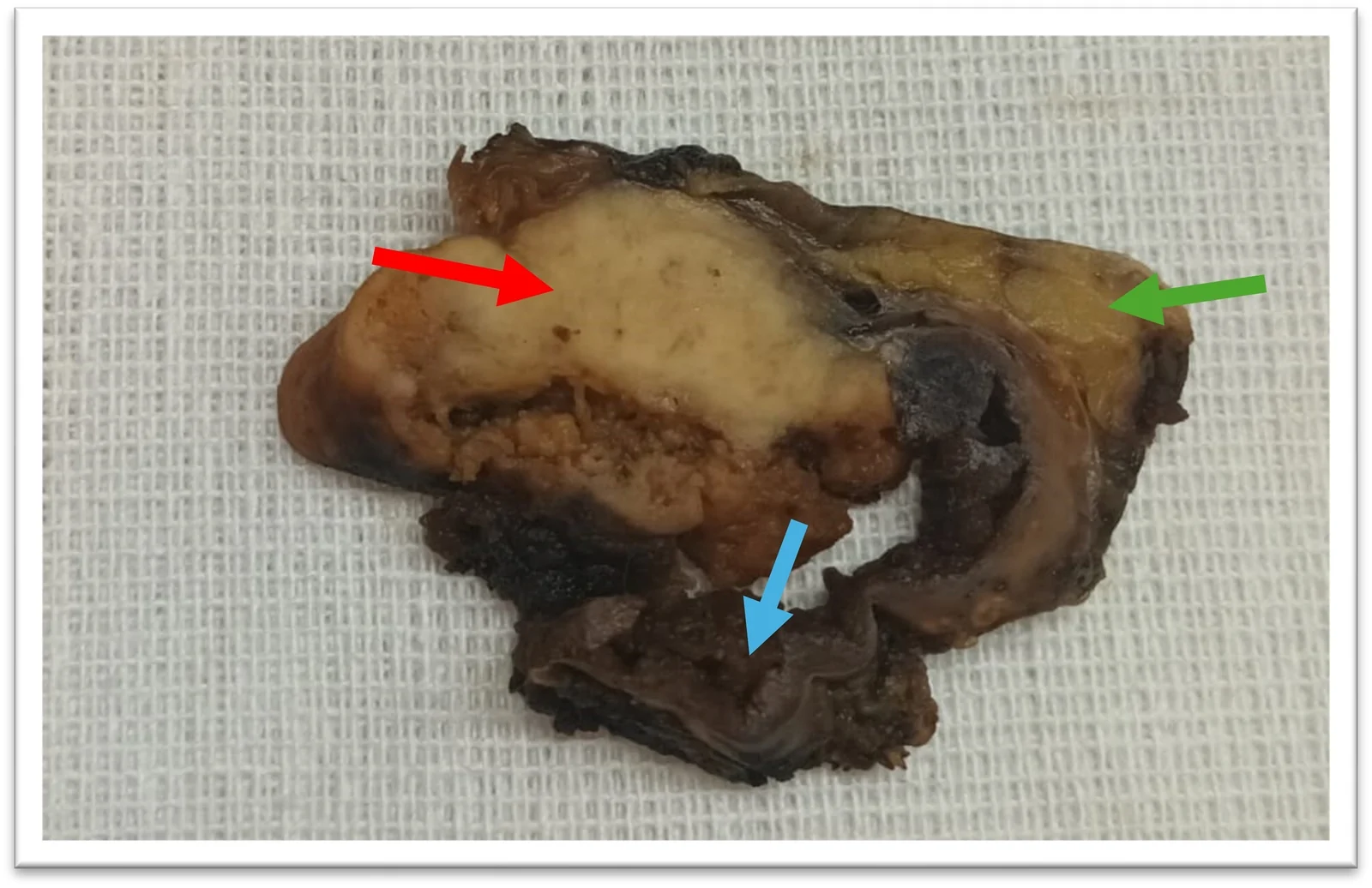
Macroscopic appearance of adrenal tumor specimen. Gross examination showing a heterogeneous mass with a brownish peripheral component (component A, blue arrow), a whitish intranodal component (component B, red arrow), and a yellowish nodular lesion adjacent to the mass (component C, green arrow).

The surgical specimen consisted of the right adrenal gland, weighting 96 g and measuring 120 × 60 × 40 mm. On the cut surface, a main lesion measuring 70 mm was observed. The lesion was entirely well circumscribed and surrounded by a thin capsule. Within this lesion, 2 different components were recognized. Peripherally, the lesion appeared soft, well delimited, and brownish (component A). An intranodal area measuring 45 mm with a whitish appearance and a firm consistency, with no clear borders with the first one, was also recognized (component B). Next to this larger mass, adjacent but not intermingled with the main lesion, a yellowish nodular lesion of 2.5 cm was observed (component C), (Fig. 4).

**Figure 5:**
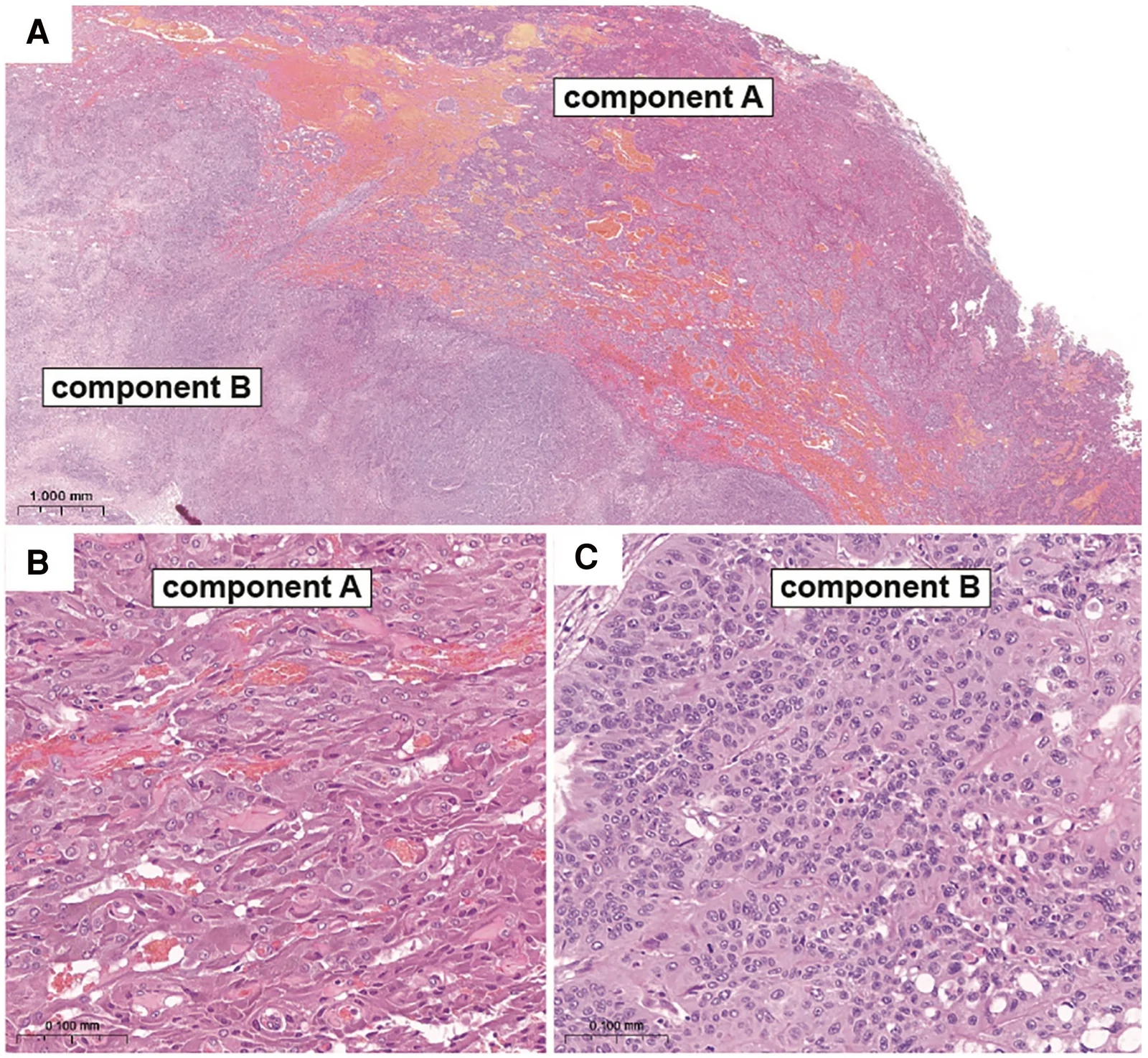
Histological evidence of tumor-to-tumor metastasis. The tumor consisted of 2 distinct neoplastic populations closely embedded in the same lesion (A): a pheochromocytoma component (component A), made of large eosinophilic polygonal cells in a trabecular arrangement (B), and a squamous cell carcinoma component (component B) composed of sheets of poorly differentiated carcinoma cells with focal keratinization (C).

Histologically, the tumor tissue corresponding to the component A was composed of large cells with an eosinophilic cytoplasm and a nesting/trabecular growth pattern. Cells of the intranodular whitish component (component B) were also large, with clear cytoplasm, highly pleomorphic nuclei, and a more solid or sheet-like growth pattern. Some focal areas of squamous differentiation were also seen (Fig. 5). Instead, the yellowish nodule (component C) showed the typical pattern of adrenocortical adenoma (not shown in the Figures).

**Figure 6:**
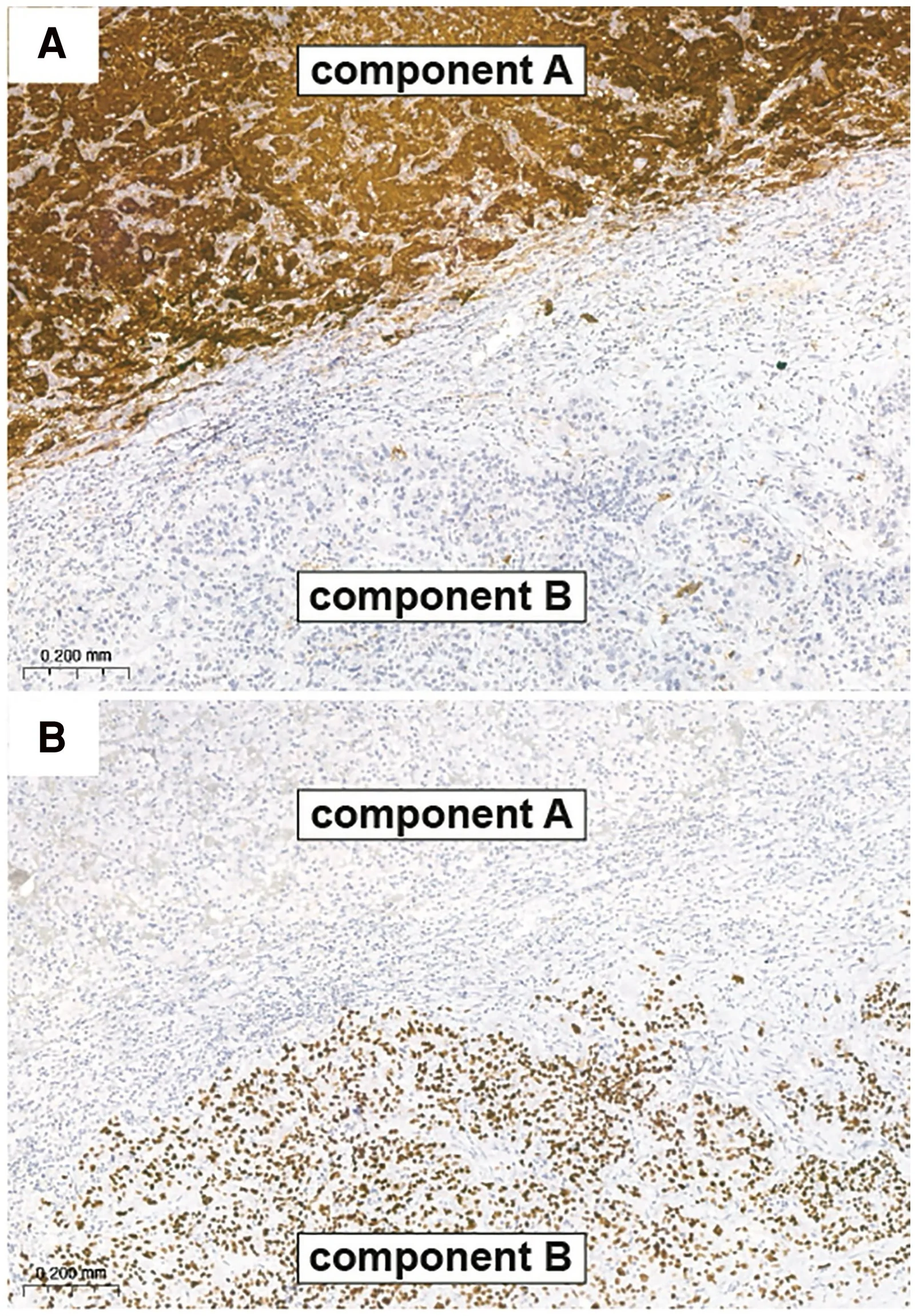
Immunohistochemical characterization of tumor components. Immunophenotyping was mutually different in the 2 tumor components merging within the same main adrenal lesion. Chromogranin A (A) was strongly and diffusely positive in the pheochromocytoma component (component A) and negative in the squamous cell carcinoma component (component B); p40 (B) showed the opposite result, being negative in the pheochromocytoma component and diffusely positive in the nuclei of the squamous cell carcinoma component.

Immunohistochemistry revealed 2 different and mutually exclusive patterns. Component A was strongly positive for the neuroendocrine marker chromogranin A (CgA, clone DAK-A3) but was completely negative for p40 (clone DAK-p40), whereas component B had an opposite immunohistochemical profile (Fig. 6). GATA-binding protein 3 (GATA3, clone L50-823) was positive in both components, but with a stronger intensity in component B. Succinate dehydrogenase subunit B (SDHB) immunohistochemistry (clone EPR10880) was positive in both components with a strong and diffuse granular cytoplasmic pattern, proving a preserved protein expression. The proliferation index (Ki67, clone MIB1) was <1% in component A and about 60% in component B. These findings established the diagnosis of a TTM of lung squamous cell carcinoma within a pheochromocytoma.

Following the histopathological and immunohistochemical findings, family history was further investigated, and no known family history of pheochromocytoma, paraganglioma, gastrointestinal stromal tumor (GIST), or other SDHx-related neoplasms emerged.

## Discussion

Tumor-to-tumor metastasis is a rare finding in patients bearing multiple tumors. According to Campbell et al, the definition of TTM requires fulfillment of the following criteria: (1) the presence of more than 1 primary tumor; (2) the host tumor must be a true neoplasm, either benign or malignant; (3) the metastatic neoplasm must be a true metastasis with established growth within the host tumor and not the result of embolization of tumoral cells or contiguous growth (the so-called “collision tumor”); (4) metastases to the lymphatic system in the presence of preexisting lymphoreticular malignancies must be excluded [8].

In the present case, histopathological confirmation of the pulmonary primary lesion was not available, which should be acknowledged as a limitation of the report with respect to the full application of the first criterion. Nevertheless, the diagnosis of TTM was supported by the coexistence of 2 distinct tumor populations within the same encapsulated adrenal lesion. Specifically, a well-defined pheochromocytoma component and a morphologically and immunohistochemically distinct squamous cell carcinoma component were identified within the same host tumor, without evidence of a separate adjacent mass, supporting true metastatic growth rather than a collision tumor phenomenon.

Although a lung biopsy performed under adequate alpha-adrenergic blockade could theoretically have provided definitive histopathological confirmation and contributed to more tailored management, a conservative diagnostic approach was adopted after multidisciplinary evaluation of the patient's clinical condition and procedural risk.

Although the pathophysiology of TTM is still unclear [9], lung cancer is the most common metastatic donor, whereas pheochromocytoma is among the rarest recipient tumors [1-3]. Hereditary or highly malignant tumors appear to be more prone to TTM. In addition, high vascularity and the expression of adhesion molecules and chemotactic factors, including integrins, cadherins, and chemokines, may facilitate metastatic seeding within recipient tumors [1, 2].

Over the last decades, the availability of multiple radiotracers has enabled a more individualized approach to functional imaging, allowing the selection of the most appropriate modality according to tumor biology and clinical context, particularly in diagnostically challenging cases. However, updated guidelines regarding the localization of pheochromocytoma and paragangliomas (PPGL) have not been published in recent years, and imaging strategies are often guided by available evidence and local expertise.

In the present case, according to the last available guidelines [10, 11], the biochemical diagnosis of pheochromocytoma was established through the assessment of plasma and urinary fractionated metanephrines, followed by a tumor staging using a CT scan. Given this diagnosis, metastatic pheochromocytoma represented a reasonable diagnostic possibility. Functional imaging with both ^18^FDG-PET and ^68^Ga-DOTA-TATE-PET was subsequently performed to stage the neoplastic lesions and refine the diagnostic hypothesis. In our patient, ^68^Ga-DOTA-TATE was used as tracer; however, a recent systematic review of functional imaging for PPGL highlighted that even if ^18^F-fluorodihydroxyphenylalanine (^18^F-FDOPA) and ^68^Ga-somatostatin analog (^68^Ga-SSA) are both sensitive for localizing PPGL, ^18^F-FDOPA is the most sensitive modality for primary pheochromocytoma and may perform similarly to ^68^Ga-SSA in metastatic disease [12].

Although ^18^F-FDG PET is no longer considered a first-line imaging modality for PPGL localization, it still plays a relevant complementary role in the diagnostic work-up, especially in tumors suspected of aggressive behavior or dedifferentiation [13]. In our patient, ^18^F-FDG PET demonstrated uptake in both the adrenal and pulmonary lesions, whereas ^68^Ga-DOTA-TATE PET showed intense uptake confined to the adrenal mass. Importantly, discordant functional imaging patterns may also occur in metastatic PPGL. Previous studies have shown that some metastatic lesions may exhibit reduced or absent ^68^Ga-SSA uptake despite strong tracer avidity in the primary tumor or in other metastatic sites, likely reflecting inter-lesional heterogeneity in somatostatin receptor expression, tumor differentiation, and biological aggressiveness [14]. Therefore, discordant findings on ^18^F-FDG and ^68^Ga-DOTA-TATE PET cannot, by themselves, completely exclude metastatic pheochromocytoma. Nevertheless, in the present case, the discrepant imaging pattern, together with the subsequent histopathological characterization, supported the presence of 2 biologically distinct neoplasms and highlighted the complementary value of combined functional imaging in complex diagnostic scenarios.

This case highlights the importance of thoroughly characterizing synchronous lesions and avoiding premature attribution of all findings to metastatic disease. In our patient, metastatic pheochromocytoma initially appeared plausible; however, histopathology demonstrated 2 distinct neoplasms. Although rare, TTM should be considered in the differential diagnosis to avoid underestimation of the phenomenon and inaccurate staging.

## Learning points

- Tumor-to-tumor metastasis should be considered in patients with multiple synchronous tumors and discordant clinical, radiological, or pathological features.
- Pheochromocytoma is a rare host tumor in TTM, with lung carcinoma being the most frequent metastatic donor.
- Discordant findings on functional imaging using different radiotracers (eg, ^18^F-FDG vs ^68^Ga-DOTA-TATE) may suggest the presence of biologically distinct tumors rather than metastatic spread from a single primary malignancy.
- Accurate characterization of coexisting lesions is essential to avoid misdiagnosis, incorrect staging, and inappropriate management.

## Data Availability

Original data generated and analyzed during this study are included in this published article.
